# A structure-guided approach for protein pocket modeling and affinity prediction

**DOI:** 10.1007/s10822-013-9688-9

**Published:** 2013-11-09

**Authors:** Rocco Varela, Ann E. Cleves, Russell Spitzer, Ajay N. Jain

**Affiliations:** 1 Certara L.P, St. Louis, MO USA; 2 Helen Diller Family Comprehensive Cancer Center, University of California, San Francisco, San Francisco, CA USA; 3 DataStax, San Mateo, CA USA; 4 Department of Bioengineering and Therapeutic Sciences, University of California, San Francisco, San Francisco, CA USA

**Keywords:** QMOD, QSAR, Surflex, MM-PBSA, Affinity prediction, Random forest

## Abstract

Binding affinity prediction is frequently addressed using computational models constructed solely with molecular structure and activity data. We present a hybrid structure-guided strategy that combines molecular similarity, docking, and multiple-instance learning such that information from protein structures can be used to inform models of structure–activity relationships. The Surflex-QMOD approach has been shown to produce accurate predictions of binding affinity by constructing an interpretable physical model of a binding site with no experimental binding site structural information. We introduce a method to integrate protein structure information into the model induction process in order to construct more robust physical models. The structure-guided models accurately predict binding affinities over a broad range of compounds while producing more accurate representations of the protein pockets and ligand binding modes. Structure-guidance for the QMOD method yielded significant performance improvements, both for affinity and pose prediction, especially in cases where predictions were made on ligands very different from those used for model induction.

## Introduction

The field of predictive modeling of chemical and biological properties in medicinal chemistry has a long tradition of pure ligand-focused model induction, starting with substituent-based QSAR approaches [1], continuing with the elaboration of fragment- and descriptor-based methods [2, 3], and including physically oriented 3D QSAR approaches [4–6]. Our introduction of the Surflex QMOD method [7] continued in this vein, focusing on induction of binding site models purely from ligand structures and associated activity values. In the present study, we instead show that a hybrid strategy that *integrates* information from experimentally determined protein structures with structure–activity data produces predictive models that are more widely applicable and accurate for ligand affinity prediction. Further, the strategy produces a binding pocket model (a “pocketmol”) directly related to the physical pocket.

The core, purely ligand-based, QMOD methodology builds and tests a pocketmol in the following six steps:

1. *Initial alignment hypothesis* Two or three ligands are chosen to serve as a seed alignment hypothesis, which is derived by maximizing their mutual 3D molecular similarity. The ligands are typically chosen to be among the most active of available data and which exhibit structural variation.
2. *Training ligand alignment generation* For each training molecule, the initial alignment hypothesis is used to guide the generation of multiple poses (typically 100–200), again using 3D molecular similarity.
3. *Probe generation* The collection of aligned active training molecules (each in their multiplicity of poses) are used to guide the placement of small molecular probes that represent possible constituents of the cognate binding pocket. Each individual training ligand pose is tessellated by probes whose fine positions are optimized for intermolecular interactions. Those probes that are not redundant of previously generated probes are retained, usually resulting in several thousand such probes.
4. *Probe subset selection* A probe subset forming an initial pocketmol is chosen to optimize multiple constraints, the most important of which is that the scores of training ligands against the pocketmol are close to their experimental values. For each ligand, it is the maximal scoring pose that defines its score.
5. *Iterative model refinement* The pocketmol is refined by iteration of the following two steps. The process stops when the final optimal ligand poses yield scores that are close to the experimental values.
  - *Pocketmol refinement* The fine positions of the pocketmol probes are optimized such that the deviation of computed training ligand scores to experimental data is minimized.
  - *Ligand pose refinement* The poses of each training ligand are refined using the current pocketmol in order to identify the optimal fit.
6. *Prediction on new molecules* The final pocketmol serves as the target of a procedure very similar to docking, in which new molecules are flexibly fit into the pocketmol to seek the optimal score subject to constraints on ligand energetics. The result produces a prediction of affinity and pose along with a measure of confidence.

The QMOD procedure is algorithmically complex, combining aspects of molecular similarity [8–10], multiple-instance machine-learning [11, 6], and docking [12–14], but all steps are fully automated. We have shown that the QMOD procedure is capable of making accurate predictions across varying chemical scaffolds [7], learning non-additive structure–activity relationships [15, 16], and guiding lead optimization toward potent and diverse ligands [17].

However, there are two key areas, corresponding to steps 1 and 3 above, which are particularly challenging when making use of structure–activity data alone. The initial alignment hypothesis is poorly constrained in the case of data that are dominated by a single chemical series, especially one with significant flexibility. In such a situation, many different initial alignment hypotheses can be generated, all of which score equally well, but only one solution will correspond well to the true binding pocket. When this happens, it is possible to derive a pocketmol that is highly predictive *within* the series but where predictions are poor on molecules with divergent scaffolds [15]. In practice, making use of multiple chemical series helps ameliorate this problem, but better means to determine an initial alignment hypothesis that represents the correct absolute configuration would lead to more predictive models. The probe generation process, step 3, is also poorly constrained, proceeding blindly without knowledge of where protein and solvent may be. Given limited structure–activity data with which to select and refine probes for a pocketmol, models can arise where “walls” are placed where only solvent exists in the true binding pocket. Both of these problems were evident when inducing a model of the CDK2 binding site using a congeneric series of substituted guanines [15]. As with the alignment problem, methods that constrain the potential pocket probe configurations such that they more closely match what is physically responsible for observed activity patterns will aid in generalization.

**Fig. 1:**
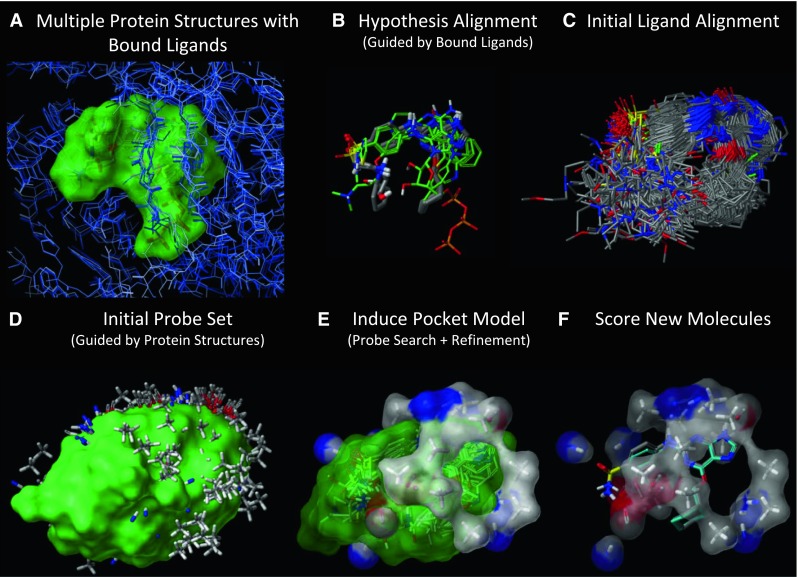
Derivation and testing of a CDK2 pocketmol using the SG-QMOD procedure: **a** a collection of multiple protein structures are aligned (bound ligands shown with a *green surface*); **b** an alignment seed hypothesis, guided both by docking and similarity to known bound ligands; **c** alignments for each training ligand are produced; **d** a large set of probes (many thousands) is created where interactions may exist; probes are filtered based on the location of similar type fragments in the protein pockets; **e** a small near-optimal set of probes (atom *colored surface*) is selected, followed by iterative probe and pose refinement; **f** new molecules are tested by flexible alignment into the final pocketmol to optimize their scores

In this work, we augment the standard QMOD procedure in two ways, both of which make direct use of experimentally determined structures of the protein target in question. The structure-guided QMOD (SG-QMOD) approach alters steps 1 and 3 of the standard procedure and is illustrated in Fig. 1. Aligned protein structures with their cognate ligands (panel a) are used to help guide the model construction process. The standard QMOD steps 1–3 correspond to panels b–d of the figure, with steps 4–5 consolidated into panel e, and the final step corresponding to panel f.

The SG-QMOD procedure makes use of the protein-ligand complexes in order to derive a more accurate initial alignment hypothesis than is possible using molecular similarity alone (panel b). Rather than relying solely upon molecular similarity, the training ligands to be used for the alignment hypothesis are *docked* into the representative crystal structures and high scoring poses are retained for each. A single pose for each is selected such that the combined mutual 3D similarity among the training ligands (including the known bound ligand poses) is maximized. This produces an alignment hypothesis that is informed both by optimal fitting with the proteins and by 3D shape and electrostatic concordance with known ligand configurations.

Additionally, the protein structures are used to *filter* the set of possible pocketmol probes produced by the standard probe generation procedure. This process *removes* probes that are not within the vicinity of similar chemical fragments in the protein binding pockets. A comparison of panels a and d in Fig. 1 reveals good overall coverage of the binding site between the protein structures in a and the initial probe set in d. Regions at the front and right-side are adequately covered by the initial rich probe set, while the hinge-binding region at the top is correctly represented by steric and polar probes, and the opening of the pocket at left remains unoccupied. This limits the initial pool to those that are spatially and chemically justifiable. Apart from these two modifications, the standard model induction and testing procedures are used.

SG-QMOD was applied to CDK2, with detailed comparisons to the standard QMOD approach, docking-based predictions, and descriptor-based QSAR modeling. Two inhibitor sets were used, the “congeneric set” consisting of substituted guanine inhibitors and the “diverse set” consisting of structurally variant inhibitors with known bound configurations. Additional comparisons included structure activity data for urokinase, Chk1, and PTP1b [18].

There were three primary results of this study. First, the SG-QMOD procedure was predictive within the congeneric CDK2 series, but SG-QMOD yielded performance similar to the purely ligand-based QMOD approach in terms of numerical accuracy. This also held for the descriptor-based QSAR predictions, with statistically indistinguishable results from the two QMOD variants. Second, for the *structurally diverse* set of molecules, the structure-guided approach was more widely applicable and accurate in both activity and pose predictions. Here, the SG-QMOD procedure yielded much better results than the purely ligand-based QMOD method, direct molecular docking, or descriptor-based QSAR. Third, for all four targets, the structure-guided procedure produced models that shared high physical concordance with the protein targets under investigation. For example, in the CDK2 case, the induced structure-guided model showed a very direct relationship with key kinase binding site elements known for their role in ligand recognition. In the urokinase case, the key interactions within the P1 recognition pocket were similarly well recapitulated. The integration of structural information provided improvements in activity prediction, bioactive pose prediction, and fidelity of induced pocketmols to experimentally determined structures of binding sites.

In addition to the methodological results, another theme emerged from the comparison of different methods. Within chemical series where the effect of substituent changes is largely additive, it is difficult to discern performance differences between computationally expedient regression-based methods, moderately expensive approaches such as QMOD, or very intensive calculations such as dynamics-based simulation approaches. However, additivity occasionally breaks down quite dramatically [15, 16], and a very common case in medicinal chemistry requires predictions on molecules quite different from those upon which a model is constructed. In these cases, stark performance differences emerge among different methods.

## Methods and data

The primary results of this study involve two sets of CDK2 inhibitors, with additional control experiments on three targets that were the subject of another study [18], all of which are described here. Procedural details of the two new algorithmic components that SG-QMOD adds to the standard QMOD protocol are also presented in this section. Additional details regarding computational and data preparation procedures presented in the “Experimental section.”

### Molecular and activity data

**Fig. 2:**
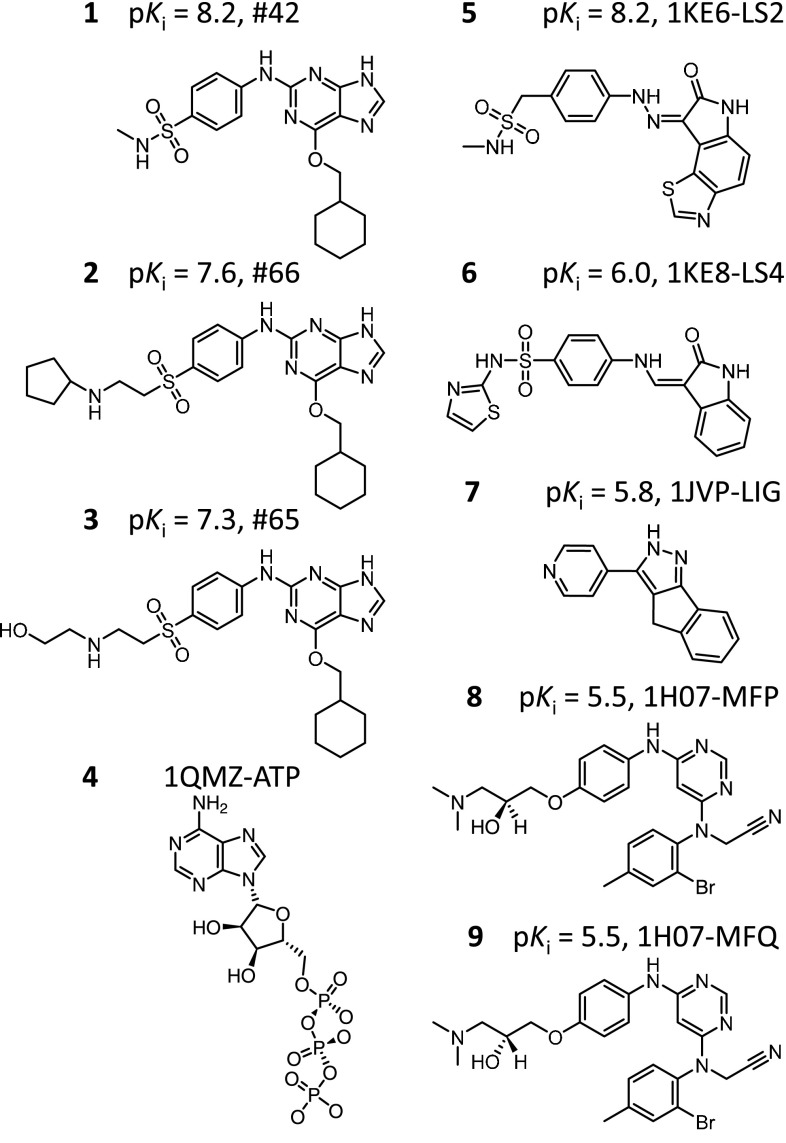
CDK2 ligands used for the alignment hypothesis. Molecules **1–3** are the top 3 most active ligands derived from the CDK2 training series used in this study. Molecules **4–9** are the bound ligands extracted from the five protein structures chosen for structural guidance (all shown with activity values were included in the training set)

For the CDK2 study, the availability of a large number of compounds within a particular chemical series was complemented by a set of inhibitors with diverse scaffolds whose bound structures were known. The congeneric series of inhibitors included 80 substituted guanine CDK2 inhibitors whose structures and activities were published from 2002–2006 [19–21]. The 80 congeners were divided randomly between 30 used for training and 50 used for testing, following an earlier report [15]. Figure 2 shows examples of the congeneric series (molecules **1–3**).

A set of 77 X-ray co-crystal structures of CDK2 were curated from Binding MOAD [22] and the RCSB Protein Data Bank [23], with assay values also gathered from BindingDB [24] where available. The co-crystal structures were organized into two groups, one for use in structural guidance during training and the other to provide a diverse set for testing. The first group had deposition dates that preceded publication of the aforementioned 80 inhibitors and included 26 structures [25]. The 26 bound ligands were all structurally different from the guanine series. From this set, five X-ray crystal structures were chosen based on binding pocket configurational diversity. These five were used for structure-guided model construction. Figure 2 shows the cognate ligands of these structures (molecules **4–9**, respectively corresponding to 1QMZ, 1KE6, 1KE8, 1JVP, and 1H07). The date cutoff was chosen such that the structures used to inform model construction would have been available at the time that the congeneric series was being investigated.

The remaining set of 52 structurally diverse compounds were used as a challenging test of the QMOD models, both for binding affinity prediction and for pose prediction. Careful inspection was carried out to eliminate inadvertent retesting of training molecules to ensure the integrity of “blind” tests. The automated curation procedure for the diverse test set was designed to identify only those ligands whose cognate PDB structure was deposited after initial publication of the guanine series. However, subsequent detailed manual inspection of the deposition records indicated that 4 of the 52 had been deposited earlier. Nonetheless, these were of varied chemical structure, and the 52 molecule test set was predominated by future compounds relative to the initial investigation of the congeneric guanine series.

A comparison to the MM-PBSA approach was also made, utilizing three targets (urokinase, Chk1, and PTP1b), originally reported by Brown and Muchmore [18]. Each data set was divided randomly between half used for training and the remaining half used for testing. The urokinase data set contained 75 ligands (37 training and 38 testing). The Chk1 set contained 123 ligands (59/57 train/test, with seven molecules not used due to structural errors or duplications). The PTP1b set contained 110 (55/55 train/test). In addition, PDB co-crystal structures with bound non-covalent inhibitors were organized for each data set to serve as a selection pool from which structures were chosen for model guidance (as with CDK2).

### Structural guidance in alignment generation

The standard QMOD procedure would make use of molecules **1–3** using molecular similarity alone. Given the common core structure and significant flexibility in some of the compounds, many high-scoring alignment solutions that are inconsistent with fitting into the CDK2 binding site are generated. Given that the SG-QMOD procedure makes direct use of experimentally determined structures to aid in determining molecular alignment, the obvious approach would be to simply dock the chosen molecules and make use of their top-scoring poses. The difficulty with this straightforward approach is that the “cross-docking” problem is well-established to be challenging for docking algorithms [26, 27]. With aggressive search procedures, using conformational variants of the protein binding sites during docking, it is possible to frequently obtain an accurate pose *among* the top scoring set, but it is often the case that the single top scoring pose is inaccurate.

Consequently, the SG-QMOD procedure combines the docking approach with molecular similarity, as follows:

1. The ligands to be used in an alignment hypothesis are docked using a standard multi-structure docking protocol [26]. The top 100 highest scoring poses for each are retained. For CDK2, molecules **1–3** in Fig. 2 were subjected to docking. Default parameters for multi-structure docking with Surflex-Dock are used [28].
2. The poses from step 1 are combined with those of the cognate ligands from the multi-structure docking. For CDK2, the cognate ligands were molecules **4–9** in Fig. 2. Molecules **1–3** have uncertainty, existing as multiple poses, but molecules **4–9** have a single pose each.
3. The pairwise 3D molecular similarities for all poses of all ligands are computed and retained. Default parameters for Surflex-Sim are used [9].
4. A brute-force optimization procedure is used to identify the single pose for each molecule which maximizes the sum of all pairwise molecular similarity values.

**Fig. 3:**
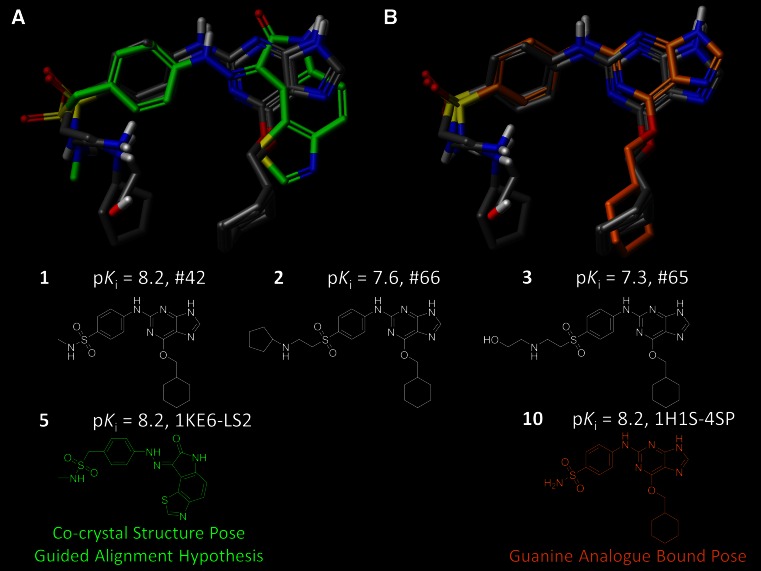
Alignment hypothesis yields conformational concordance among highly active CDK2 ligands while satisfying physical constraints of observed bound conformations: **a** the hypothesis alignment of the top three most active CDK2 ligands (**1–3**, *atom color*) with crystal structure bound pose of **5** (*green*); **b** hypothesis alignment of ligands **1–3** with bound pose of structurally related analog **10** (PDB code: 1H1S, *orange*). Compound **10** was not used during the hypothesis generation

Figure 3 shows the result of this procedure applied to the CDK2 case. Panel A shows the selected poses superimposed with one of the known bound ligands (compound **5**, PDB code 1KE6). There is high concordance among the hinge-binding polar moieties at the top-right, a matching ring orientation in the center, and matching disposition of the sulfonamide/sulfone substituents at the left. Panel b shows the same alignment, but includes the bound pose of a guanine analog *not used* for model construction (compound **10**, PDB code 1H1S). The chosen poses for training compounds **1–3** fully mirrored compound **10**.

**Fig. 4:**
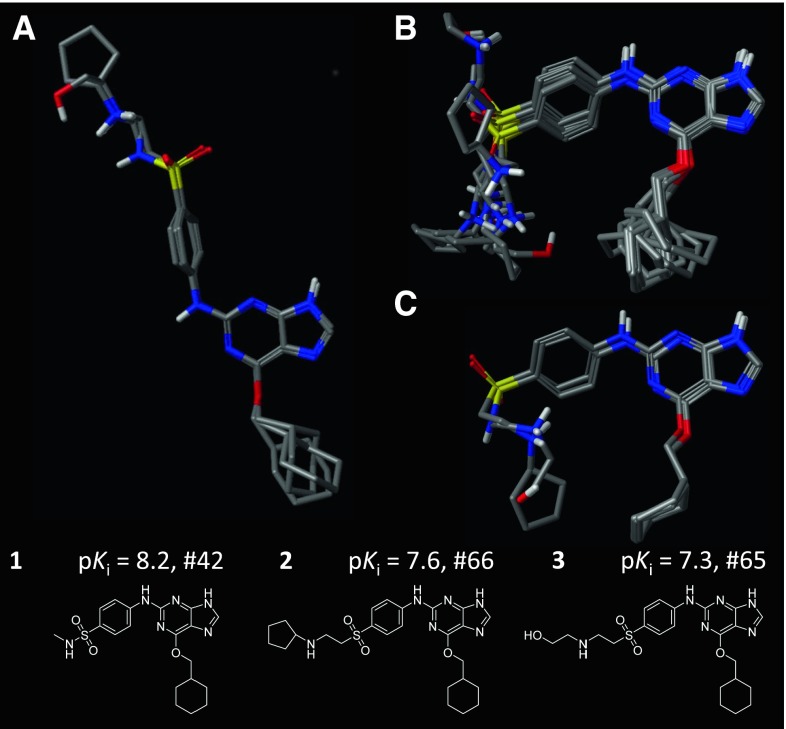
Variations of alignment hypotheses: **a** the top-scoring result of pure ligand-based hypothesis generation for molecules **1–3**; **b** the five top-scoring docked poses of the same three molecules; **c** the three poses from among 100 docked poses selected based upon mutual similarity, including the cognate ligands of structures used for guidance

Figure 4 shows the contrasting results obtained by using pure molecular similarity or pure docking. In the former case (panel a), an extended conformation of the guanine analogs was chosen, with nitrogen-substituent being flipped opposite to the oxygen-substituent. This configuration is clearly not correct in terms of the absolute geometry of such inhibitors within the CDK2 pocket (see Fig. 3). However, because the correspondence of parts among the inhibitors is reasonable, it is possible to induce a model that is predictive within this series, as will be discussed below. In the case of docking, the five highest scoring poses for each of the three ligands are shown (panel b), each of which fall within a narrow window of scores. There was some positional variation of the hydrophobic substituent, but the uncertainty in the orientations on the left-hand side were more substantial, allowing for two alternatives for the sulfonamide substituent. Within the collection of docked poses, however, there were a set of poses that were both concordant with one another and also with the bound poses of molecules **4–9** used to help guide the SG-QMOD procedure. Panel c shows these poses, which correctly disambiguate the orientation of the sulfonamide substituent as well as providing a tight initial alignment of the hydrophobic pendant group.

The SG-QMOD alignment procedure makes use of docking, but it employs molecular similarity as an additional constraint, thus allowing a more coherent set of poses to be derived. Consideration of similarity to known bound configurations of other ligands offers the type of guidance that an experienced modeler may employ when working with a target where additional experimental information exists. In the CDK2 case, poses chosen in this fashion are reasonable, based the concordance between the guanine analogs to a highly similar analog whose bound structure was known (see Fig. 3).

### Structural guidance in pocketmol probe generation

**Fig. 5:**
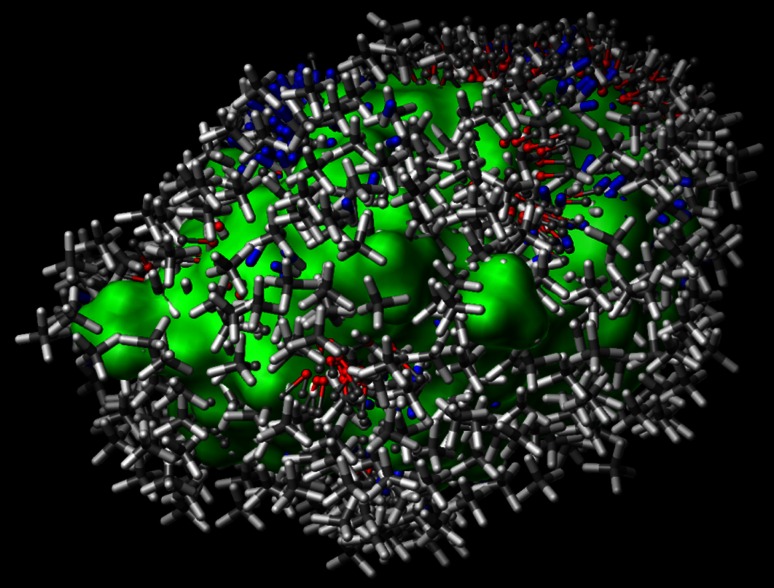
The full probe set from the standard QMOD procedure, prior to the filtering step used in the structure-guided approach

Lacking any information about the true binding site, the standard QMOD procedure simply identifies the positions of all three types of probes that can make favorable interactions with *any* part of any pose of an active training ligand. For the alignment hypothesis depicted in Fig. 3, this results in the probe set shown in Fig. 5. In contrast to the filtered set shown in Fig. 1d, the full set locates probes in places that are exposed to solvent. While it is possible to *learn* that a part of space is not part of the true pocket, this requires the presence of structural variation within a ligand training set in all such locations.

In the structure-guided procedure, the probes are filtered so that a probe will only exist in a position that is close to an atom of similar type within any of the protein pocket variants being used. In order to survive the process, a steric probe must fall within 1.0 Å of a hydrophobic atom of a protein variant, and a polar probe must fall within 2.0 Å of an atom of the same polarity on a protein variant. Comparing Fig. 1a with d, the filtered probe positions correspond to the occupancy dictated by the aligned protein structures, representing a logical “OR” among the different conformations. The process yields a set of probes that allow for variability beyond what is seen within the crystal structures used, but the probes are constrained to have some geometric support from those structures.

## Results and discussion

The target case of CDK2 offers a particularly rich example, with hundreds of protein structures available, bound to a diverse complement of inhibitors. The available structures covered significant conformational variation in the enzyme binding site. For CDK2, it was possible to assess performance of the SG-QMOD procedure on both affinity prediction and pose prediction and to compare performance with the standard QMOD procedure, direct molecular docking, and to descriptor-based QSAR predictions. The comparative performance of the methods varied considerably on the test against inhibitors of diverse chemical structures, and these results will be presented first.

Comparison of SG-QMOD to MM-PBSA was also made, making use of a comprehensive study of three targets for which data were made available [18]. In these cases, SG-QMOD performed similarly to the CDK2 case. However, structural variety among the ligand sets was more limited, reducing the power to discern comparative differences.

### CDK2: congeneric and diverse test ligands

A common situation in lead optimization efforts arises where the precise bound configuration of a series under active consideration may not be known, though there may be significant structural information on other chemical series. This situation is studied here using the congeneric set of CDK2 inhibitors coupled with structural guidance. The SG-QMOD procedure identified good initial poses for training molecules **1–3**, as discussed above, from which the alignments for the remaining molecules were generated as in the standard procedure. The SG-QMOD probe generation method made use of protein structure information to influence the pool of probes for pocket construction (Fig. 1d). From this point, the standard model induction procedure was carried out.

#### Predictive performance: within-series and beyond-series

**Fig. 6:**
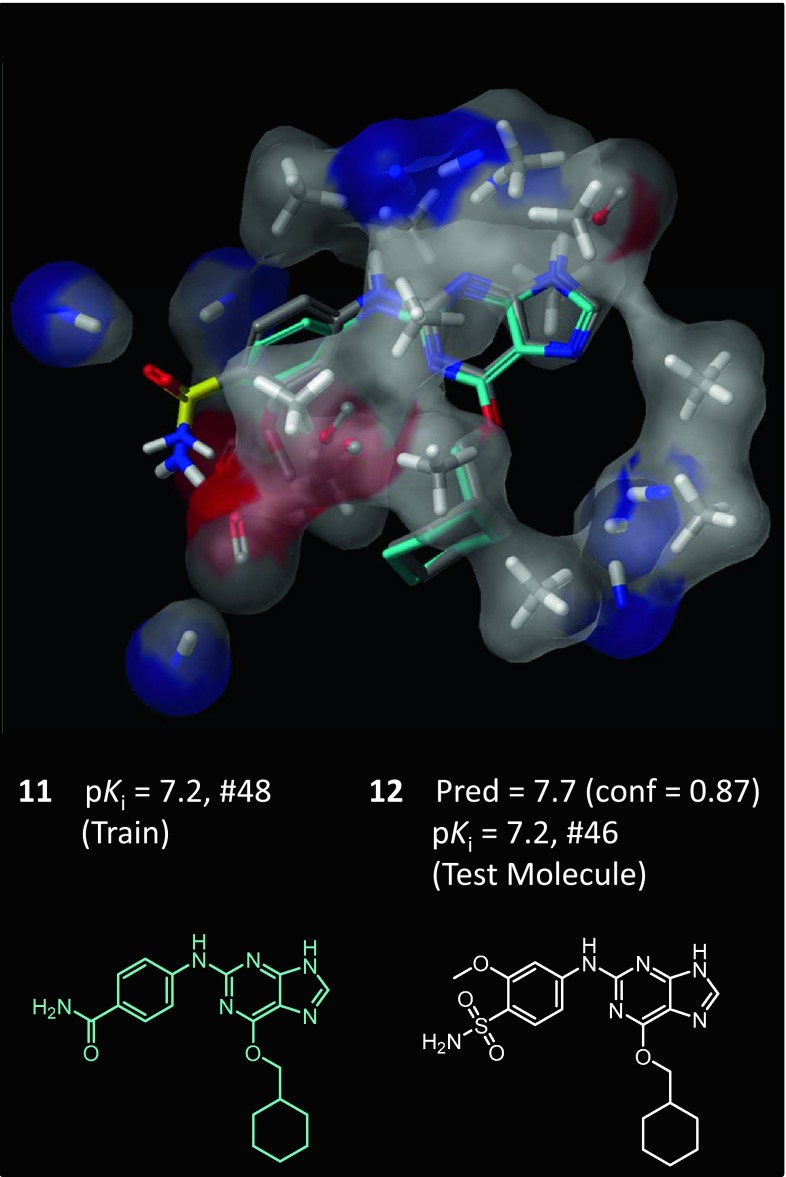
The final SG-QMOD CDK2 pocketmol is shown in thin sticks and skin, with molecule **12** (atom-*colored sticks*) in its final predicted pose. The high confidence reported for **12** derived from similarity to training molecule **11** (*cyan*). The predicted activity of **12** was just a 0.5 log unit deviation of its actual p*K*_*i*_

**Fig. 7:**
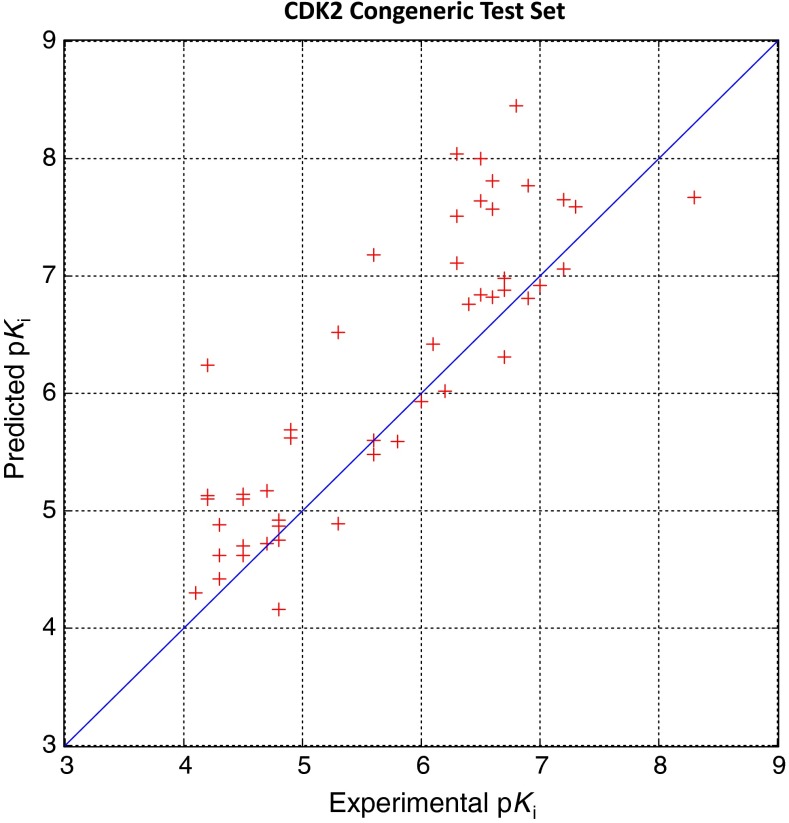
SG-QMOD produced accurate predictions within the CDK2 substituted guanine series. Activity prediction performance on the entire guanine series test set is plotted. The overall prediction error was 0.61, with a Kendall’s Tau rank correlation of 0.73 (*p* < 0.01, by permutation analysis), and an r^2^ of 0.71. Note that while, in this case, overprediction was more common than underprediction, this is not a general trend

Figure 6 shows the final pocketmol, and Fig. 7 shows prediction performance on the 50 CDK2 substituted guanine inhibitors. The model was highly predictive within this series, producing an average error of 0.61 log units and a Kendall’s Tau rank correlation of 0.73 (*p* < 0.01). The final pocketmol with the predicted pose of molecule **12** is shown. The QMOD procedure estimated confidence for a new ligand based on the similarity of predicted bound pose to that of a training molecule. Here, high confidence stemmed from training molecule **11**, and the predicted activity of compound **12** (p*K*_*i*_ = 7.7) was very close to correct (a 0.5 log unit deviation). Performance on the entire set is shown at right, highlighting the excellent correlation. For the purposes of synthetic candidate prioritization, it was notable that 7 out of the top 10 predicted test molecules appeared among the top 10 *bona fide* most potent molecules in the entire test set.

**Fig. 8:**
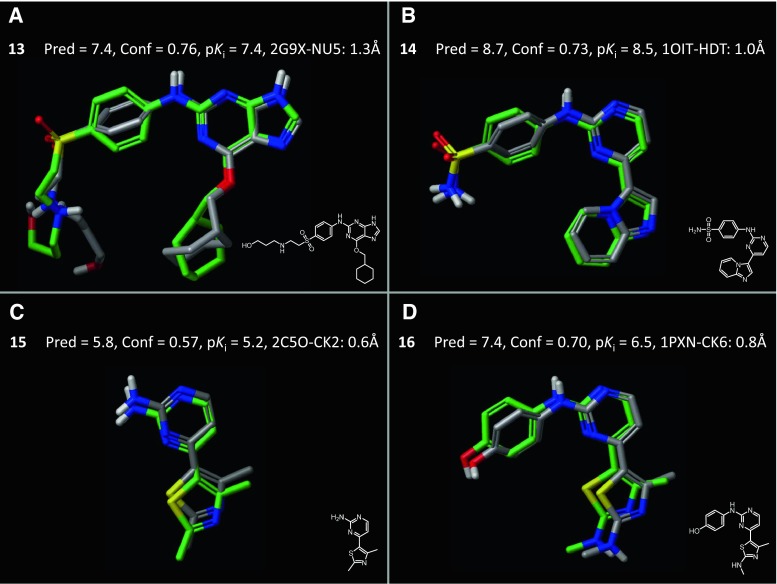
The structure-guided modeling procedure produced accurate pose and activity predictions on the diverse set of 52 CDK2 inhibitors. Panels **a** shows an example of a substituted guanine compound closely related to the training molecules, and **b**–**d** show representative examples of diverse CDK2 ligands in their predicted poses (atom-*colored*) superimposed with their crystal structure bound pose (*green*)

Another common situation requires transfer of SAR from one series to other series, in the case where a change of scaffold my be required. Figure 8 shows representative examples of predictions made on the diverse test set with the structure-guided model. Panel a contains a substituted guanine whose bound pose was determined experimentally. The predicted and experimental poses deviated only with respect to the end of the flexible tail at the left-hand side, which reaches toward a solvent-exposed area. High confidence and low error were to be expected from a close analog to those on which the model was trained. The molecule in Panel b shares direct structural similarity with respect to the left-hand-side of the training series, but the central scaffold and lower substituent are quite different. Again, though, all aspects of the prediction were accurate. Panel c shows a test ligand that deviated further still, sharing very little in common with any training molecules, which was reflected in the low confidence value, but prediction errors were still small. Panel d shows a structural elaboration of that shown in c, where the predicted improvement in pK_*i*_ was 1.6, but the actual was 1.3, resulting in an over-prediction of activity by 0.9 log units. However, the indication of substantial improvement was correct, as was the predicted bound configuration of the inhibitor.

Predictive performance for activity and pose is summarized for the diverse inhibitor set in Table 1 (top). Overall, SG-QMOD yielded an average activity prediction error of 1.1 (units of pK_*i*_) and an average RMSD 1.8 Å, with a Kendall’s Tau rank correlation of 0.27 (*p* = 0.01). As expected, the model performed systematically better on ligands for which confidence was higher. At confidence levels of 0.7 and above, average RMSD was 1.2 Å, and the ranking was nearly perfect (Tau = 0.85, *p* < 0.01).

**Table 1:** SG-QMOD and docking results for 52 structurally diverse CDK2 inhibitors

Confidence	NMols	p*K*_*i*_ range	Average error (p*K*_*i*_)	Kendall’s Tau	*p* value	RMSD (Å)
*SG-QMOD*						
0.7	9	5.0–8.5	0.7	0.85	<0.01	1.2
0.5	39	4.5–9.9	1.0	0.30	<0.01	1.6
All	52	3.5–9.9	1.1	0.27	0.01	1.8
*Direct docking*						
0.7	9	5.0–8.5	2.2	0.43	0.11	2.5
0.5	39	4.5–9.9	2.0	0.26	0.02	2.4
All	52	3.5–9.9	1.8	0.24	0.01	2.4

Given that experimentally determined protein structures are being used in the SG-QMOD procedure, it is natural to wonder how *direct* use of the structures for affinity and pose prediction performs. It is particularly apt here, because QMOD makes use of the same scoring function as Surflex-Dock in quantifying the intermolecular interactions between ligand and either pocketmol (for QMOD) or protein binding site (for Surflex-Dock). Beginning with similar conditions used for the SG-QMOD approach (i.e. the same five protein crystal structures with bound ligands), we docked the set of 52 diverse inhibitors to assess the performance of naïve and direct use of structural information. Table 1 (bottom) summarizes the results, broken down by groups of molecules as defined by the SG-QMOD confidence levels. In all cases, SG-QMOD produced much lower absolute errors.

Two aspects were somewhat surprising. First, the docking approach produced better than expected results, with statistically non-random rankings of affinity in the two larger molecule subsets despite high absolute errors. SG-QMOD performance was still more robust, with the quality of the rankings increasing among the more confidently predicted molecules, even producing statistically significant ranking results within the smallest group. Second, and perhaps more surprisingly, the *pose prediction* performance of SG-QMOD was better than the pure docking approach as well. We speculate that the process of identifying parsimonious structural explanations for ligand affinity helped identify key features for ligand binding, which in turn led to improvements in pose identification and ranking.

#### Relationship of the induced pocket model to the CDK2 binding site

In the foregoing, we have discussed the effects of integrating protein structural information on accurate prediction of ligand activity and bioactive pose within the protein pocket. Another important attribute is the physical relationship of the induced model with the protein binding pocket. The pocketmol is not a literal re-representation of a binding pocket, being instead generally more sparse, reflecting the physical characteristics that best explain the activities of known ligands. Pocket flexibility can be represented in the pocketmol structure with multiple alternative probes, and complex electrostatic fields can be represented by the combination of multiple probes, even of multiple types.

**Fig. 9:**
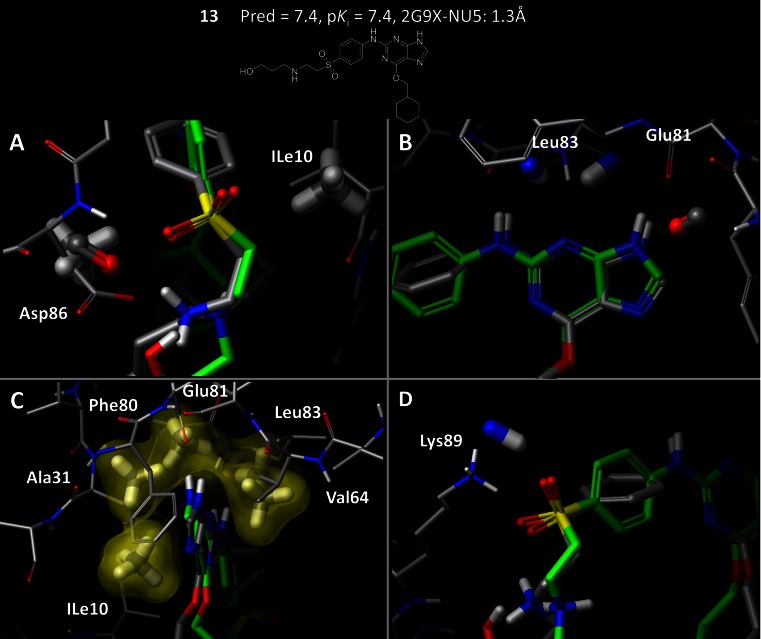
The induced pocketmol from the structure-guided procedure matches key physical characteristics of the binding pocket. The predicted pose of compound **13** (*gray*) is shown with the bound pose (*green*) to provide a frame of reference. Panels **a**–**d** provide detailed snapshots of key regions of the binding pocket that are well represented by the pocket model. **a** Polar aspects of Asp86 are captured by an acceptor probe and two hydrophobic probes provide matching physical constraints on the *right* and *left-side* of the pocket. **b** The backbone carbonyl of Glu81 is modeled by an acceptor probe and the NH group of Leu83 is captured by two donor probes. **c** Hydrophobic probes (shown with a *transparent surface*) model the physical shape of the buried pocket region defined by ILe10, Ala31, Val64, and hinge residues 80–83. **d** Lys89 is represented by a donor probe at the opening of the pocket

To the extent that a pocketmol accurately reflects true binding pocket geometries, there will be broader compatibility with diverse ligands exhibiting significant structural novelty. Figure 9 highlights the physical relationship between the model (probes shown in thick sticks) and the crystallographically determined binding site of 2G9X (thin sticks) with a bound ligand (green). Key interaction points on the hinge binding region are well represented by the induced pocketmol. Electrostatic interactions provided by Asp86 are modeled by an acceptor probe, and two hydrophobic probes flanking the right and left sides of compound **13** closely match physical constraints provided by Asp86 and ILe10 (Fig. 9a). The backbone carbonyl of Glu81 is modeled by an acceptor probe and the NH of Leu83 is represented by donor probes (Fig. 9b). Panel c shows an outward view of the buried portion of the pocket highlighting structural concordance between a series of hydrophobic probes and the arc-like shape of the pocket defined by ILe10, Ala31, Val64 and hinge binding residues 80–83. The electrostatic interaction between Lys89 and the sulfone group is modeled by a donor probe (Fig. 9d).

**Fig. 10:**
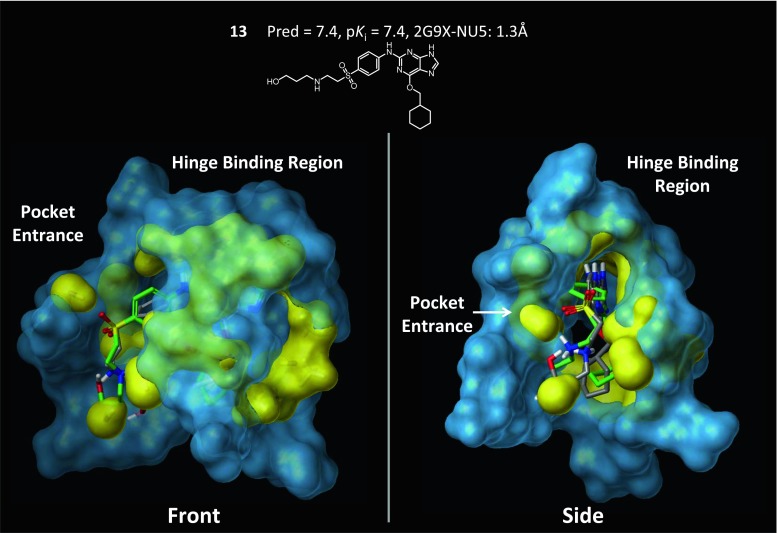
The structure-guided QMOD procedure produces a pocketmol that captures the overall shape and electrostatic elements of the CDK2 binding pocket. The 2G9X binding pocket is shown with a *blue surface* and with the final pocketmol as a surface. The predicted pose of compound **13** (atom-*colored sticks*) is shown with its bound pose (*green sticks*)

In addition, the pocketmol provided a highly concordant physical shape of the binding pocket, accurately characterizing the overall structural configuration. Figure 10 illustrates these shared characteristics. The front view highlights congruence between the pocketmol (yellow) and the binding pocket (blue), with coverage of the perimeter of the binding cavity. The side-view shows a rotated and clipped view of the pocketmol and protein, highlighting similarity in overall volume between the pocketmol and binding pocket. Note that the flexible “tail” of **13**, where a deviation exists between the predicted and bound poses, protrudes into solvent. The charged secondary amine, however, interacts with the pocketmol probe that correctly represents the contribution of Asp86 (see Fig. 10a). In cases such as in CDK2, where a lysine sidechain is in a solvent-exposed position, the degree of importance for binding affinity can be difficult to assess. The SG-QMOD approach allows the ligand activity data to help make that determination in a fashion that is compatible with the known structural information.

To further quantify the degree of concordance between the pocketmol and the CDK2 binding pocket, we examined the relationship between CDK2 pocket atoms that were particularly important for ligand binding and the closeness of matching SG-QMOD pocketmol probes. Recall that the probe generation procedure produces several thousand probes, which in the SG-QMOD procedure are filtered to retain those that are close to matching protein atoms. The filtered probe set, from which the learning process begins, contained a matching probe for essentially every protein atom that was proximal to the binding pocket. So, the extent to which the final composition and fine positioning of the pocketmol probes corresponded to important protein pocket atoms was driven by finding a configuration that fit the activity data. All intermolecular atomic contacts of the diverse 52 ligand set (in their crystallographic poses) and the aligned set of five CDK2 protein structures were identified. The CDK2 binding pocket atoms were partitioned into two groups: (1) pocket atoms with close contact (surface distance of < 0.5 Å) to 90 % or more of the ligands; and (2) those with at least one close contact but not more than with 10 % of the ligands.

For each of the two groups, for each protein pocket atom, the distance to the nearest pocketmol atom of matching type was computed. This resulted in two distributions of distances: one corresponding to those pocket atoms that participated in close contacts with the 52 test ligands very frequently (e.g. the hinge-binding atoms) and the other corresponding to the pocket atoms in infrequent contact with the ligands. The two distributions of pocketmol probe distances were statistically very different (*p* ≪ 1 × 10^−6^ by Kolmogorov–Smirnov). In particular, the protein atoms with high-participation had a matching pocketmol probe within 2.0 Å 70 % of the time. The low-participation atoms had a matching pocketmol probe less than 30 % of the time.

The pocketmol induction process made use of 30 guanine analogs and the six non-guanine compounds from Fig. 2 along with corresponding activity data. The 52 compounds used to partition the protein atoms for this analysis were from the blind test set of diverse compounds. The relationship between a protein atom having frequent interaction with these molecules and being more likely to have a matching pocketmol probe arose from the model induction process. It seems likely that part of the reason for improved performance in pose prediction for SG-QMOD compared with direct docking stemmed from the enrichment within the pocketmol for atoms that are important for binding.

### Comparison to standard QMOD

The particular substituted guanine chemical series was the subject of a previous QMOD study, where no structural guidance was used [15]. Here, a direct comparison to the structure-guided model was made by constructing a standard QMOD model for CDK2 using only the substituted guanine series (i.e. not including compounds **4–9** in training). Figure 4a depicts the corresponding alignment hypothesis. The standard model yielded numerical predictive performance that was statistically equivalent to what was observed for the structure-guided procedure on the 50 compound guanine-based inhibitor test set (mean prediction error of 0.5 with a Kendall’s Tau rank score of 0.73). This is not especially surprising, as many QSAR approaches can perform well *within* a chemical series, especially if the separation of training and testing is done through random selection.

However, when we considered performance on the 52 chemically diverse CDK2 inhibitors with known bound configurations, we observed much improved performance using the structure-guided approach than using the control procedure (results summarized for SG-QMOD in Table 1). The standard ligand-based QMOD model yielded rank correlation performance indistinguishable from random on the full set of 52 inhibitors, with average RMSD > 3.7Å, but the SG-QMOD model yielded significant rank correlation and mean RMSD of 1.2 Å. For the control QMOD model, only when the confidence threshold was raised sufficiently to exclude all but 6 test compounds did rank correlation become significant (Tau = 0.82, mean error = 1.2), but the mean RMSD was unchanged. Overall, the structure-guided model predicted 46 % of inhibitors with errors < 0.75 log units, 61 % with errors < 1.0, and 78 % < 1.5. The standard ligand-based procedure, yielded, respectively: 10, 16, and 30 %.

### Comparison to descriptor-based QSAR

Given that the QSAR problem itself can be addressed by a range of methods, it is interesting to ask how well simpler approaches perform. As in our previous study involving gyrase [17], here we applied descriptor-based QSAR using the random forest learning (RF) algorithm (see “Experimental section” for details).

#### RF as a general QSAR control

In the previous study, which focused on iterative temporal model refinement, there were two interesting observations regarding the RF versus QMOD approaches. First, the RF approach performed quite well in a purely numerical sense with respect to prediction accuracy, identifying comparable numbers of active compounds to the QMOD approach. Our expectation had been that because the QMOD model exhibited strong concordance to the structure of the biological target, i.e. that it was making predictions for the right reasons, that it would yield notably better performance in identifying active ligands over the iterative experiment. The second surprise had to do with the characteristics of the potent compounds uncovered by the two methods. The primary difference between the QMOD “winners” and those of the RF approach was not in number but in kind. Those for the QMOD approach had much greater structural diversity, representing quite different ways to effectively inhibit the gyrase target. The RF approach, by contrast, identified a collection of highly self-similar winners.

In the report that introduced QMOD [7], a similar test procedure was employed as we have here, restricting training to a single chemical series of amino-tetralin 5HT1a ligands, but tests were carried out on diverse ligands, many of which had been discovered much later. At the time of that study, we did not employ the RF descriptor-based approach as a standard control. However, to better understand the value of this type of control, we applied RF on the 5HT1a set, training on 20 ligands and testing on two sets. The first set contained 35 compounds structurally related to the training set, and the RF approach produced statistically indistinguishable rank performance to the QMOD results (Kendall’s Tau of 0.39 for the former and 0.38 for the latter, both having *p* values < 0.01). The second set contained 32 diverse compounds, of which the activities of 17 were supported by multiple independent assays. For the diverse set, the RF approach yielded performance no better than random on the full set (n = 32, Tau = 0.17, *p* = 0.17) or on the subset (n = 17, Tau = 0.28, *p* = 0.09). QMOD yielded statistically significant rank performance on both sets, respectively producing Tau of 0.29 and 0.51 (*p* = 0.03 and *p* < 0.01). The RF approach can be surprisingly accurate and robust, and the cases in which it yields poor performance represent interesting challenges for predictive activity modeling.

#### RF applied to CDK2

The RF approach was applied here, training on the 30 guanine analogs and testing on the diverse 52 inhibitor set. The results here largely paralleled what we found in the previous gyrase study. However, given the increased structural diversity of the test set, the overall predictive performance for the RF method was lower than for QMOD (results summarized in Table 2). Overall, for the 52 inhibitors, the RF approach yielded Kendall’s Tau of 0.21 (*p* = 0.05), with average error of 1.2 pK_*i*_ units (comparable values for QMOD were Tau of 0.27 (*p* < 0.01) and mean error of 1.1). The differences were more substantial as the relative confidence of each method was considered. For QMOD, its standard confidence measure was applied (described earlier). For the RF approach, the analogous computation was made, equating confidence for each test ligand to the most similar training ligand by Tanimoto similarity of the descriptor fingerprint used. For the least confident half of the test set, the RF method produced random rankings: Tau = 0.12 (*p* = 0.26) and mean error = 1.4. But for the QMOD method, overall set performance was nearly matched by performance on the bottom half: Tau = 0.34 (*p* = 0.03) and mean error = 1.0. Conversely, for the top quintile of confidence, the RF method produced marginal performance: Tau = 0.38 (*p* = 0.13) and mean error = 1.1. But QMOD performed well, yielding Tau = 0.87 (*p* < 0.01) and mean error of 0.65.

**Table 2:** Comparison of QMOD to RF on 52 diverse CDK2 ligands

Method and subset	N	Kendall’s Tau	*p* value	Error (p*K*_*i*_)	RMSD (Å)
*SG-QMOD*					
Top confidence quintile	10	0.87	<0.01	0.65	1.3
Top half	26	0.30	0.03	1.1	1.5
Bottom half	26	0.34	0.03	1.0	2.2
All	52	0.27	<0.01	1.1	1.8
*Random forest*					
Top confidence quintile	10	0.38	0.13	1.1	NA
Top half	26	0.35	0.02	1.1	NA
Bottom half	26	0.12	0.27	1.4	NA
All	52	0.21	0.05	1.2	NA

#### Structural diversity of identified winners

**Fig. 11:**
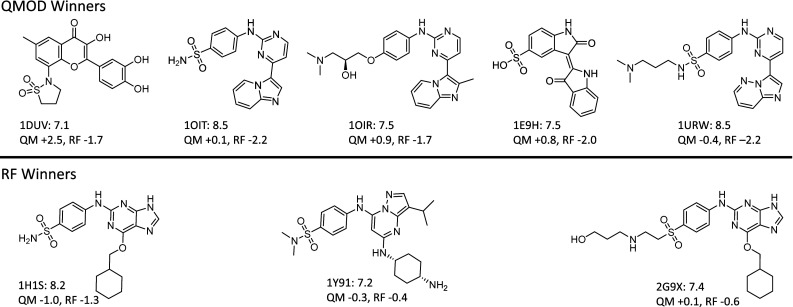
The structure-guided QMOD procedure top five predictions included five molecules with pK_*i*_ ≥ 7.1 (*top row*). The RF procedure’s top five predictions included three such potent compounds (*bottom row*). The PDB codes and experimental pK_*i*_ are shown in the labels, along with the magnitude and sign of the prediction error by the two procedures

Of the top five compounds predicted to be most active, QMOD correctly identified five with pK_*i*_ ≥ 7.1, and the RF approach identified just three such compounds. More importantly, as seen in Fig. 11, the relatively potent compounds identified by the QMOD approach were of much more varied chemical structure than seen for the RF approach. None of the five SG-QMOD “winners” were of the substituted guanine class that dominated the training set. For the RF winners, two of the three were variations on this scaffold. Generally, for the RF approach to make a prediction at the extremum of activity, the structure in question must share significant topology with training compounds. The QMOD approach, by contrast, is agnostic to topology, and is sensitive only to the degree that the new molecule fits into and complements the pocketmol. Here, we see that the RF predictions dramatically underpredict activity for the SG-QMOD winners (by about 2.0 log units). In contrast, the SG-QMOD predictions were more accurate than those made by RF on all of the RF winners.

It is also worth noting that, while numerical prediction of affinity has utility, accurate prediction of binding modes for novel ligands during lead optimization also has substantial value in guiding the design process. For the three RF winners, SG-QMOD produced an average RMSD of 1.5 Å. For its own five winners, the SG-QMOD approach produced a mean RMSD of 1.7 Å, establishing to some degree that the predictions were being made for the “right” reasons. The relative value of pose prediction compared with affinity prediction is both subjective and project-dependent. However, as compounds must be contemplated with divergent scaffolds from those with available activity data, methods such as the SG-QMOD approach, which reach toward causal inference rather than simple correlative inference, can produce better results across multiple criteria.

### Comparison to simulation-based affinity prediction

The foregoing comparisons to direct molecule docking and to descriptor-based QSAR addressed the question of how well widely-used and easily applied structure-based and ligand-based methods would fare in comparison to the SG-QMOD approach. In the case of straightforward docking, the results were not altogether surprising, given the known limitations of docking approaches for binding affinity estimation. In the case of descriptor-based QSAR, the results paralleled our previous observations [17]. Direct comparison to more complex simulation-based approaches were not explored on the CDK2 data set. These represent an entirely different level of computational complexity, and such approaches also require deep methodological expertise. Highly experienced practitioners of such methods report that, while accurate for some systems, they can produce poor results for others without obvious explanations. Apart from methodological and computational complexity, each of which can be overcome, the inability to know, a priori, which systems will be amenable to simulation-based approaches remains a serious practical challenge. Chodera et al. [29], Gilson and Zhou [30], and Blaney [31] provide excellent reviews of the state-of-the-art, theoretical basis, and limitations for such methods.

**Fig. 12:**
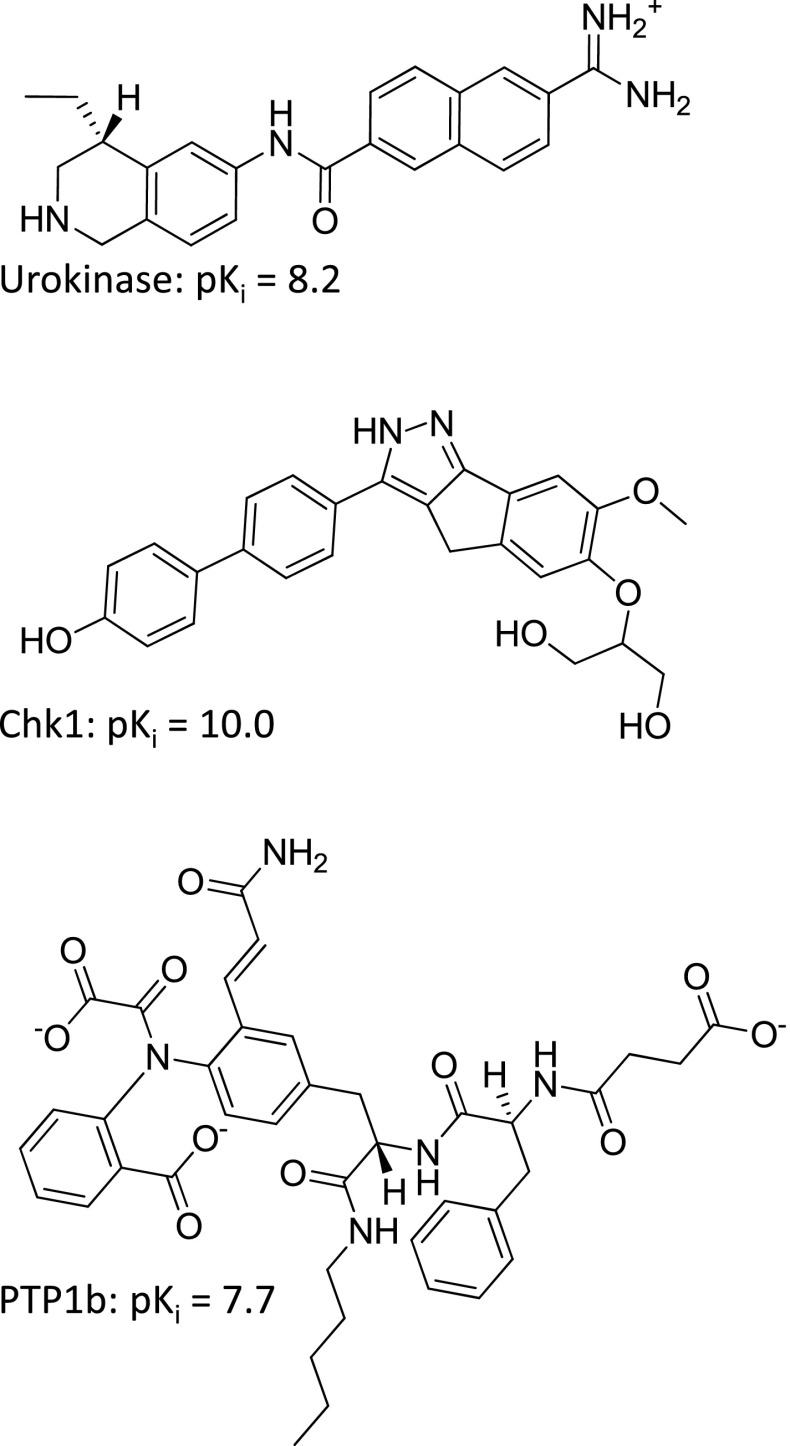
Examples of potent compounds from the three targets of the Brown/Muchmore [18] MM-PBSA study

One nicely done study applying MM-PBSA by Brown and Muchmore [18] demonstrated results of sufficient quality to be potentially useful for guiding lead optimization. Figure 12 shows representative examples of the ligands for urokinase, Chk1, and PTP1b. There are a number of considerations that make direct comparison of the SG-QMOD and MM-PBSA results complicated. First, the latter method does not require any training, being instead a simulation-based approach that had been automatically applied. For this comparison, the molecular data was partitioned randomly into 50/50 sets for training and testing of SG-QMOD. We avoided leave-one-out cross-validation in order to make a more realistic test. Nonetheless, training on a proportion of data offered an advantage to the QMOD method. Second, results from the MM-PBSA approach represent best-case performance in two respects. The experimentally determined bound configuration for each ligand was used in the simulations, thus obviating any dependence on uncertainty in ligand pose. To varying extents, but particularly in the case of PTP1b, with very large and flexible ligands, this represents a significant advantage for the simulation approach. Also, in reporting standard errors, the authors linearly re-scaled the actual prediction values in order to minimize RMSE, owing to the sharp difference in slope and intercept for predicted versus experimental pK_*i*_ values.

**Fig. 13:**
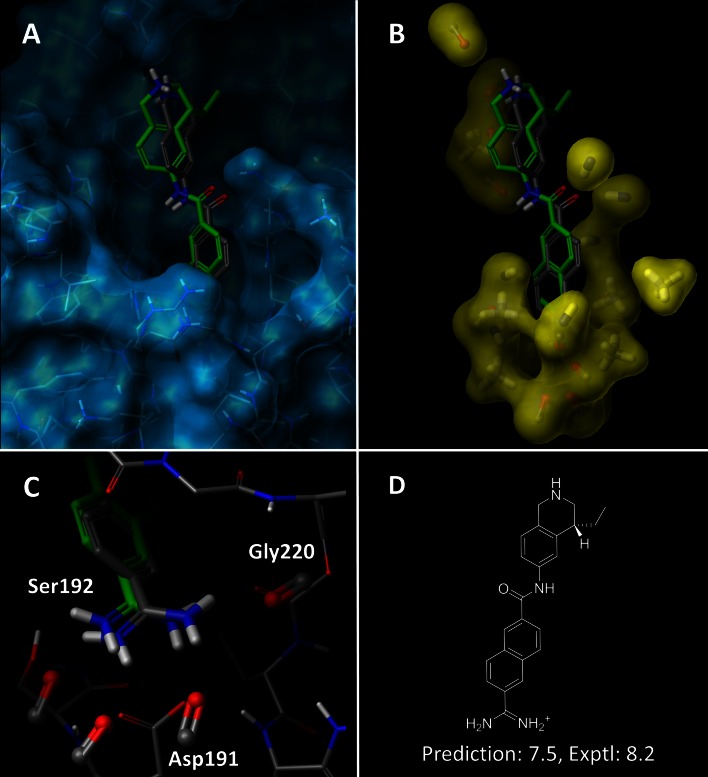
SG-QMOD model for urokinase: **a** predicted pose (atom-*colored sticks*) compared with the crystal bound pose (*green sticks*) of ligand 497 in the context of the protein binding pocket (PDB ID: 1OWD, *blue skin* with thin sticks); **b** the same ligand poses within the QMOD pocket model (*yellow skin* with sticks); **c** detail of the pocket model acceptor probes interacting with the amidine portion of the molecule; **d** 2D schematic of test molecule 497

The SG-QMOD procedure described for the CDK2 example above was applied to the three targets (see “Experimental section” for details). Figure 13 shows the resulting urokinase pocketmol aligned with the urokinase protein structure, depicting the predicted pose of the inhibitor from Fig. 12. The pocketmol accurately represents the shape and polar characteristics of the binding pocket while making an accurate prediction of binding activity and pose of a test molecule. The primary aspect of pose uncertainty for this relatively rigid compound was the orientation of the amide substituent. The prediction arising from fitting to the pocketmol was close to correct, driven by a favorable interaction with an acceptor probe (top left of panel b) that corresponds well with one from the protein (see panel a). The RMSD for this compound was 1.3 Å. The learned representation of the deep P1 pocket structure (panel c) shows direct correspondence between the acceptor probes of the SG-QMOD model and analogous functionality within the protein. The probe placement and orientation is driven by the requirement to produce an energetic field, which, given the functional form and parameterization of the intermolecular scoring function, produces the correct activity values at the extremum of molecular score with respect to pose. This physical abstraction allows better reproduction and prediction of activities across a wide range of new molecules than can be achieved through direct use of docking.

Automated application of the MM-PBSA method and the SG-QMOD method gave rise to the results summarized in Table 3 (numbers in parentheses are 95 % confidence intervals for the statistics). With the caveat that the QMOD approach makes use of training data and that the application of MM-PBSA benefited from having no uncertainty in the poses of any molecules, the comparison based on the correlation metric shows very similar performance. For urokinase, QMOD performed slightly better, and for PTP1b the MM-PBSA approach performed slightly better. However, the differences were marginal. Comparison of the RMSE values is less informative, because the values for MM-PBSA were linearly re-scaled to minimize the residual squared errors. Without such a correction, the QMOD error magnitudes were much lower. Overall, given that the MM-PBSA results represent a best-case scenario for a simulation-based technique, it is encouraging that the realistic application of SG-QMOD produced comparable results that could be beneficial in a real-world lead-optimization application.

**Table 3:** Comparison of SG-QMOD to MM-PBSA on three targets

Method and target	N	Tau	*p* value	RMSE	R
*SG-QMOD*					
Urokinase	38	0.79	<0.001	0.66 (0.51–0.81)	0.86 (0.79–0.92)
Chk1	57	0.50	<0.001	1.09 (0.88–1.27)	0.70 (0.55–0.81)
PTP1b	55	0.48	<0.001	0.99 (0.79–1.22)	0.66 (0.44–0.80)
*MM-PBSA*					
Urokinase	75	–	−	0.69	0.78
Chk1	123	–	–	0.89	0.72
PTP1b	110	–	–	0.66	0.83

## Conclusions

The core Surflex-QMOD methodology has been validated in prior studies [7, 15–17]. The significance here relates to the integration of information from protein structures within the QMOD model induction process. Within the congeneric chemical series studied here, the SG-QMOD procedure performed similarly to the purely ligand-based QMOD approach with respect to prediction error magnitude and activity ranking. As we have observed before, such models were unable to correctly identify absolute bound configurations given data from a single chemical series. By contrast, the structure-guided procedure offered substantial advantages, producing models that reliably mimicked the true protein binding sites. For structurally diverse test molecules, such models provided much more accurate ligand activity ranking and pose prediction. The structure-guided models identified key characteristics important for ligand binding and yielded excellent pose predictions, better even than those produced by docking methods. It is important to note that the advantages observed were often only detectable when the model testing procedure followed real-world application scenarios.

Construction of the diverse CDK2 testing set was done temporally, with the molecules on which predictions were to be made having been reported *in the future* compared with training molecules. The advantages for SG-QMOD were most strongly observed in this test scenario, without a guarantee that a highly similar near neighbor would exist within the training set used for model induction. We believe that assessment of QSAR methods using techniques such as leave-one-out cross-validation on data sets of single congeneric series is of limited value. In our previous report, exploring iterative QMOD model refinement over time, we observed strong effects on the trajectories of discovered molecules [17]. Application of random forest learning with molecular descriptors was accurate in a numerical sense, but yielded pools of active compounds with shallow structural diversity compared with those produced by application of QMOD. Here, we observed a similar effect, but also saw an advantage in terms of pure predictive performance of SG-QMOD over RF on the diverse CDK2 test set.

The QMOD method is clearly quite dependent on the degree to which the initial ligand alignments match the true relative configurations of the training ligands, especially regarding generalization to compounds with divergent scaffolds. Inclusion of diverse ligands in training or augmentation with bound ligand information, as done here, improves performance. The method is also highly dependent on the degree of variation within the available training data. The models cannot know what they have not seen, though this is an issue in common across all QSAR methods. Given protein structure information, it is possible to constrain the search space of possible models to ones that do not deviate very far from physical expectations. However, the search space is still very large, and multiple pocketmol solutions still exist. Arbitration among them is done empirically. The most reliable method has been to select the model with the property that the optimal training poses of molecule pairs close in activity exhibit high 3D molecular similarity (this is a quantitative measurement of model parsimony).

Still another area for exploration is the degree to which, given molecules with low to mid-range potency for training, that the method is able to help “climb the hill” toward more potent molecules. Given the strong dependence on molecular alignment, this may prove to be challenging, though active selection of structurally novel compounds (irrespective of their predicted potency) offers a strategy that was beneficial in the case of bacterial gyrase [17]. With large and diverse data sets that span long time periods becoming increasingly available, we believe that it will be possible to systematically investigate this question.

Apart from conclusions about the methods reported here, there are four features that we would hope to see become a standard part of future assessments of QSAR methodology. First, questions should be asked about predictive performance over time, including characterization of the structural diversity of identified active compounds. Second, test molecules should be included that have significant structural distance from training molecules (this is often a natural consequence of temporal segregation). Third, because the problem of relative alignment between chemically different series is challenging in real-world lead-optimization, assessment of pose prediction should become a part of 3D-QSAR investigations. Last, robust and easy-to-apply methods such as random forest learning using fingerprint-based molecular descriptors should be employed as a standard control. We believe that this combination will help reveal the degree to which any method will be shown to be useful in the wild, where chemical structural novelty is often an absolute requirement in molecular design.

With respect to the methods reported here, experimentally determined protein structural information can clearly be profitably exploited to augment ligand structures and associated activities. In this work, we have shown how to construct QMOD pocketmols in a manner that is constrained to make use of direct structural information. The clear extension to the method is to dispense with the pocketmol formalism and instead to refine the structures of an ensemble of aligned protein binding pockets. The goal would be to use the refined ensemble directly, with a simple docking-based scoring scheme, for affinity prediction. This requires a simple extension to the multiple-instance learning formalism, where in addition to the ligands having the potential for variation, the binding site itself would also be represented as variants. The score for a ligand given an ensemble of protein pocket variants would simply be the one resulting from the optimal fit to *any* of the variants. Such an approach fits in the gap between the approach described here and the purely physics-based simulation-oriented methods.

In any event, the results reported here encourage the development and use of hybrid methods that maximize information gleaned from different sources, including both biophysical information on protein structure and information from experimental determination of ligand activities.

## Experimental section

### Ligand preparation

Ligand molecular data sets were described within the “Methods and data” section. All assay values were converted into molar pK_*i*_ units (9.0 being equivalent to a K_*i*_ of 1 nM). The standard Surflex procedure was used to protonate, ring-search, and minimize the ligands. This resulted in up to five conformations per inhibitor, with protonation as expected at physiological pH. These prepared structures were used for all subsequent procedures.

### Computational procedures

The QMOD procedure is automatic, requiring no human choice points. For this work, default parameters were used, employing Surflex-QMOD version 1.5. There were two significant algorithmic variations investigated here, compared with that reported in the most recent study [17]. First, an initialization protocol was added that incorporates multi-structure docking and data integration that uses bound ligand poses to guide the generation of an alignment hypothesis. Second, a procedure was added that filters an initial probe pool using guidance from multiple aligned protein structures (see Fig. 1, panels a, d). All protein structures used in this study were pre-processed using standard procedures for structure preparation and mutual alignment [32, 33].

In the CDK2 study, five representative structures were chosen by k-means clustering from a pool of 26 protein structures. The top 3 most active CDK2 training ligands (see Fig. 2 molecules **1–3**) were docked against these five structures using the standard Surflex-Dock multi-structure docking protocol [26, 34–36]. Multi-structure docking was carried out using Surflex-Dock v2.7, with an option to retain up to 100 docked poses per ligand. The *particular* pose used in QMOD model construction for each of these three docked ligands was chosen to maximize the pairwise similarity among the docked poses and the 6 co-crystal ligands (see Fig. 2 ligands **4–9**). Molecular similarity calculations were carried out using Surflex-Sim v2.7.

For the MM-PBSA comparison an analogous approach was carried out. For each data set 5 representative co-crystal structures were chosen from their respective selection pools using k-means clustering. These representative co-crystal structures were used for model guidance. For the PTP1b study the top 2 most active training ligands were docked, and poses were generated and selected as described above. In the urokinase and Chk1 cases the training ligands were well represented by the selected co-crystallized ligands used for model guidance, and so the co-crystal bound poses were chosen for subsequent model setup.

The compounds with poses chosen as described above were used as the alignment target in the standard QMOD procedure to produce initial alignments for all training molecules in all cases. The probe pool was initiated using the standard tessellation procedure which has been described in our earlier work [7]. This large set of molecular probes surrounds the initial alignments of the training ligands, where each probe makes a near-optimal interaction with at least one active ligands pose. Initial probe pools were then filtered using the representative protein structures chosen for model guidance. Every probe was evaluated against similar atom types (e.g. donors, acceptors, hydrophobic) on the protein structures. Probes that were within a predefined minimal distance (i.e. 2.0 Å polar, 1.0 Å hydrophobic) to similar type atoms comprised the filtered probe pool used for model induction.

The procedure for producing a de novo pocketmol requires a single command from a simple setup file that produces a script. The script runs a sequence of QMOD commands that generate initial alignment hypotheses, full alignments of training ligands, and final pocketmols. The setup file contains information on path names to training ligands and their activities, which ligands to use for hypothesis generation, and modifications to default parameters for model building if desired. For the generation of pure ligand-based models with no structural guidance, the standard procedure was employed. For the structure-guided models, the augmentations just described replaced the normal alignment hypothesis generation step and filtered the initial probe pool. All other steps remained as in the standard protocol. By default, three models are generated, each using different probe densities. In all cases, the model with the highest reported parsimony was selected for blind testing and structure evaluation.

Surflex-Dock v2.7 was also employed as a control procedure for comparing rank ordering and pose prediction accuracy of the 52 diverse CDK2 ligands. For these computations, multi-structure docking was carried out as described above, using the same five protein crystal structures used for the SG-QMOD approach.

Random forest learning was applied as previously described. The random forest technique is an ensemble classification approach that constructs multiple decision trees using a random sampling approach to minimize generalization errors [3, 37, 38]. We used the random forest method implemented in version 4.6-2 of the randomForest package for the R software environment (version 2.12.2). Unity 988-bit fingerprints were generated using SYBYL-X 2.1 (both programs from: Certara, L.P., 9666 Olive Blvd. Suite 425, St. Louis, MO 63132 USA). The model training and molecule testing procedure paralleled that used for QMOD, making use of default parameters for the RF learning procedure. To mimic the confidence procedure, we calculated Tanimoto similarity scores between testing and training molecules using the Unity fingerprints. This provided an analogous metric for measuring confidence using features employed by the classifier.
